# The miRacle in Pancreatic Cancer by miRNAs: Tiny Angels or Devils in Disease Progression

**DOI:** 10.3390/ijms17060809

**Published:** 2016-05-26

**Authors:** Zuhair Hawa, Inamul Haque, Arnab Ghosh, Snigdha Banerjee, LaCoiya Harris, Sushanta K. Banerjee

**Affiliations:** 1 Cancer Research Unit, VA Medical Center, Kansas City, MO 64128, USA; zzhawa@hotmail.com (Z.H.); aghosh2@kumc.edu (A.G.); lacoiya.harris@va.gov (L.H.); 2 Division of Oncology, Department of Internal Medicine, University of Kansas Medical Center, Kansas City, KS 66205, USA; 3 Department of Anatomy and Cell Biology, University of Kansas Medical Center, Kansas City, KS 66205, USA; 4 Department of Pathology, University of Kansas Medical Center, Kansas City, KS 66205, USA

**Keywords:** microRNA, pancreatic cancer, OncomiR, tumor suppressor, PanIN, cancer stem cells, epithelial to mesenchymal transition, TS-miR

## Abstract

Pancreatic ductal adenocarcinoma (PDAC) is an aggressive malignancy with increasing incidence and high mortality. Surgical resection is the only potentially curative treatment of patients with PDAC. Because of the late presentation of the disease, about 20 percent of patients are candidates for this treatment. The average survival of resected patients is between 12 and 20 months, with a high probability of relapse. Standard chemo and radiation therapies do not offer significant improvement of the survival of these patients. Furthermore, novel treatment options aimed at targeting oncogenes or growth factors in pancreatic cancer have proved unsuccessful. Thereby, identifying new biomarkers that can detect early stages of this disease is of critical importance. Among these biomarkers, microRNAs (miRNAs) have supplied a profitable recourse and become an attractive focus of research in PDAC. MiRNAs regulate many genes involved in the development of PDAC through mRNA degradation or translation inhibition. The possibility of intervention in the molecular mechanisms of miRNAs regulation could begin a new generation of PDAC therapies. This review summarizes the reports describing miRNAs involvement in cellular processes involving pancreatic carcinogenesis and their utility in diagnosis, survival and therapeutic potential in pancreatic cancer.

## 1. Introduction

Pancreatic ductal adenocarcinoma (PDAC) is an aggressive malignancy with increasing incidence and high mortality. PDAC is among the ten most commonly diagnosed cancers and is the fourth leading cause of cancer related death in the United States with a five-year survival rate of approximately 3–6 percent and a median of survival rate of 2–8 months [1]. Although the standard of care in advanced pancreatic cancer is improved through targeted therapies and personalized medicines [2,3], prognosis and treatment of PDAC still remains unsatisfactory. Due to the impalpable, asymptomatic and invasive nature of the disease, diagnosis of PDAC at an early stage is impossible. PDAC typically presents with metastasis at the time of diagnosis. Unfortunately, no curative treatment is available for advanced stages of the disease.

Surgical resection is the only potentially curative treatment of patients with PDAC, followed by chemotherapy. However, because of the late presentation of the disease, only 15 to 20 percent of patients can go in for surgery. The patients who undergo the procedure are likely to relapse, but the average survival of resected patients is between 12 to 20 months [4]. Standard chemo and radiation therapies do not offer significant improvement of survival for these patients. New treatments targeting known oncogenes, or growth factors, in pancreatic cancer such as K-Ras, vascular endothelial growth factor (VEGF) and epidermal growth factor (EGF)/epidermal growth factor receptor (EGFR) have mostly failed and do not provide survival benefits [5,6]. Therefore, it is important to identify biomarkers that can detect early stages of this devastating disease with high sensitivity and specificity. Among these biomarkers, microRNAs (miRNAs) have supplied a profitable recourse and become an attractive focus of research.

The microRNAs (miRNAs) are non-coding, small RNA molecules about 19–22 nucleotides in length. MiRNAs are post-transcriptional regulators that bind to the 3′-untranslated region (3′-UTR) on target mRNAs, usually resulting in translational repression and gene silencing [7]. Many miRNAs have been shown to play a vital role in either progression or suppression of cancer [8,9,10,11]. Recent studies have shown that miRNAs are key regulators of PDAC initiation and progression, and these events could be mediated by modulating proliferation, apoptosis, metastasis, angiogenesis, chemosensitivity, stemness and radiosensitivity [2,12,13,14,15,16,17,18,19]. The importance of miRNAs in PDAC has been highlighted by numerous studies that have shown the abnormal expression of different miRNAs can lead to chronic pancreatitis, different grades of pancreatic intraepithelial neoplastic (PanIN) and PDAC [20,21,22,23,24]. All these studies provide significant insight into altered cellular features such as growth, invasive and metastatic behavior of PDAC cells and have established a close relationship between miRNAs and PDAC progression. Recent data revealed that a comprehensive knowledge of the atypical expression of these miRNAs and reestablishment of their normal expression patterns could produce a favorable therapeutic method and help establish targeted approaches for the preclusion of this disease. These aberrantly expressed miRNAs may function either as oncogenic miRNAs (oncomiRs) (Table 1) or tumor suppressor miRNAs (TSmiRs) (Table 2), and they appear to play important roles with respect to the initiation, progression and metastatic growth of distal organs in PDAC. Thus, the possibility of intervention in the molecular mechanisms of miRNA regulation could begin a new generation of pancreatic cancer therapies. This review summarizes the recent reports describing miRNAs’ involvement in cellular processes involving pancreatic carcinogenesis and their utility in diagnosis, survival prognosis and therapy.

## 2. Biosynthesis of miRNAs

**Figure 1:**
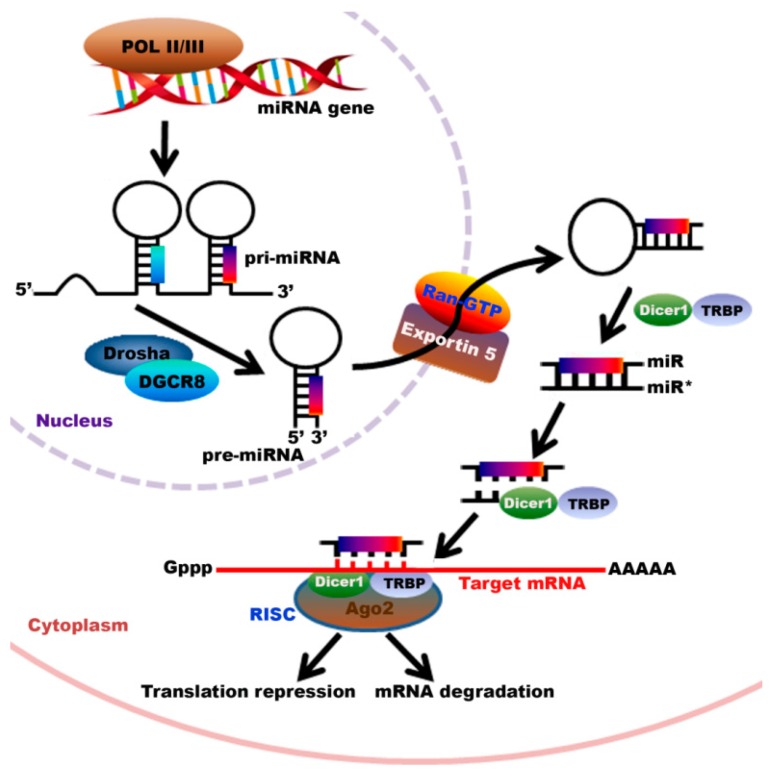
Biosynthesis of miRNAs. The miRNA is first transcribed within the nucleus by RNA polymerase II and II to generate a long primary miRNA (pri-miRNA), which is cropped by the Drosha/GDCR8 complex into precursor-miRNAs (pre-miRNAs). These pre-miRNAs are then transported to the cytoplasm through the interaction with nuclear transport receptor Exportin-5 and nuclear protein Ran-GTP. Once in the cytoplasm, the pre-miRNA is further processed by DICER1 and TAR-binding protein (TRBP) into a double stranded ~22 nucleotide product comprised of the mature miRNA guide strand and the miRNA* passenger strand. The miR* passenger strand is cleaved by Ago2 and the mature miRNA is then incorporated into the RNA-induced silencing complex (RISC) to target the 3′-untranslated region of the target mRNA.

The biosynthesis of miRNAs, or the process of making miRNAs, is a tightly regulated multistep process that takes place both in the nucleus and cytoplasm [80]. In the nucleus, the miRNA gene is first transcribed by RNA polymerase II or III to produce a long primary miRNA (pri-miRNA) characterized by hairpin structure, which is 5′-capped and 3′-polyadenylated [81]. The pri-miRNA is cropped by the Drosha/GDCR8 complex into a ~60–100 nucleotide hairpin structure termed the precursor-miRNA (pre-miRNA) [82]. These pre-miRNA molecules are then transported to the cytoplasm through the interaction with nuclear transport receptor Exportin-5 and nuclear protein Ran-GTP where it is further processed by DICER1. A second RNase III endonuclease, together with its catalytic partner, TAR-binding protein (TRBP), cleaves the pre-miRNA into a double stranded ~22 nucleotide product comprised of the mature miRNA (guide strand) and the miRNA* (passenger strand). The miR/miR* duplex is then loaded into a multicomponent complex, the RNA-induced silencing complex (RISC), constituted of at least TRBP, DICER1 and one Argonaute-2 (Ago2) [7,83]. The miR* passenger strand is cleaved by Ago2 while the miR serves as a guide for target recognition to either degrade the mRNA or cause translational suppression, depending on the level of complementarity between the miRNA and its target mRNA (Figure 1).

## 3. OncomiRs in Pancreatic Cancer

The oncomiRs are the cluster of microRNAs known to contribute to the tumorigenic process through the regulation of genes associated with cell growth and differentiation. Multiple oncomiRs have been identified thus far and were found to be actively involved with pancreatic cancer development. Six of them are described below:

### 3.1. MicroRNA 21 (miR-21)

*MiR-21* is located in the 3′-UTR of *VMP1* (vacuole membrane protein 1) gene, also known as transmembrane protein 49 (*TMEM49*), at chromosome 17q23.2 [9]. Multiple studies have suggested a relationship between aberrant expressions of miRNA and human pancreatic cancer [84]. MiR-21 is one of the most studied miRNAs in cancer, and its overexpression is associated with increased proliferation, invasive phenotypes and resistance to chemotherapeutic drug gemcitabine (Gemzar), creating the worst overall survival probability in pancreatic cancer patients [85,86,87,88,89]. The overexpression of miR-21 is associated with human PanIN grade, with peak production occurring in PanIN-2/3 lesions and regulated by K-Ras^G12D^ and EGFR in PDAC-derived cell lines [90]. MiR-21 binds with the mRNA of phosphatase and tensin homolog (PTEN), a known tumor suppressor, and represses its translation, thereby reducing its tumor suppressive action on cell proliferation of pancreatic tumor cells [25,91,92]. Increased miR-21 expression has been associated with activation of PI3K/Akt/mTOR pathway, which promotes cell survival, proliferation and progression in pancreatic cancer cells [26,93]. The invasive phenotypes of pancreatic cancer cells are promoted by miR21 via indirect regulation of matrix metalloproteinases (MMPs), as well VEGF [26,87]. A recent study showed that miR-21 is induced by hypoxia in pancreatic cancer via hypoxia inducible factor-1 α (HIF-1α) upregulation and allows cells to avoid apoptosis in a hypoxic microenvironment [94]. Thereby, miR-21 overexpression in pancreatic cancer cells is an impelling cause of cancer progression and suggested to be a potential prognostic biomarker of the patients with PDAC.

### 3.2. MicroRNA 221 (miR-221)

OncomiR miR-221 is located ~700 bp from miR-222 on the chromosome Xp11.3 [30]. Several studies have reported that miR-221 is upregulated in human primary pancreatic cancer tissues or human pancreatic cancer cell lines compared to normal pancreas tissues and pancreatic duct epithelial cells, respectively [29,84,95,96,97]. Overexpression of miR-221 has also been reported in the mouse pancreas with PanIN lesion [95]. MiR-221 promotes cell proliferation via binding with 3′-UTR of p27kip1, a tumor suppressor and a member of the Cip/Kip family of cyclin-dependent kinase (CDK) inhibitors [28,31]. MiR-221 is vital for the platelet-derived growth factor (PDGF)-mediated epithelial-mesenchymal transition (EMT) phenotype, migration and growth of pancreatic cancer cells by down-regulating tricho-rhino phalangeal syndrome type 1 (TRPS1) and p27 [31]. Moreover, the pancreatic cancer patients with high miR-221 expression had comparatively shorter survival than those with lower expression, suggesting that miR-221 could be a prognostic factor for worst survival of patients [29].

### 3.3. MicroRNA-155 (miR-155)

The miR-155 locus is located in chromosome 21q21 within a region known as the *B-cell integration cluster* (*BIC*) gene [24]. MiR-155 is overexpressed in a variety of solid, human tumors such as breast cancer [98,99,100,101], lung cancer [102,103,104], thyroid tumor [105,106] and prostate cancer [107], as well as pancreatic cancer [34,85,108,109,110]. These studies illustrate that miR-155 plays a crucial role in tumor development, diagnosis and prognosis. Pancreatic cancer cell migration, invasion and metastasis can be regulated by miR-155 via targeting 53-induced nuclear protein 1 (TP53INP1) [33,34] or by regulating the suppressor of cytokine signaling 1 (SOCS1) through the STAT3 signaling pathway [33,34,98,111]. Moreover, miR-155 negatively regulates Mut L homolog 1 (MLH1), a prognostic determinant in several cancers, including pancreatic cancer, and expressions were revealed in the K-Ras^G12D^ transgenic mouse model of PC [112,113,114,115,116,117]. Mechanistically, K-Ras activation upregulated miR-155 expression through MAPK and NF-κB pathway, and miR-155 promoted reactive oxygen species (ROS) stress via inhibiting FOXO-3a expression in pancreatic cancer [110]. Overexpression of miR-155 is correlated with poor prognoses of pancreatic cancer [113]. Collectively, these studies demonstrate that miR-155 is an important oncomiR in the development as well as aggressive behavior of pancreatic cancer.

### 3.4. MicroRNA 10b (miR-10b)

MiR-10b is located between HOXD4 and HOXD8 on chromosome 2q31.1 and is particularly associated with metastatic behaviors of cancer cells [35]. MiR-10b was first identified to promote tumor metastasis in breast cancer [36], which is regulated by a matricellular protein CCN5 via HIF-α-TWIST signaling pathway [118]. Subsequently, involvement of miR10b with tumor invasive potential in nasopharyngeal carcinoma [119,120], glioma [121,122], acute myeloid leukemia [123], esophageal cancer [124], colon cancer [125], neurofibromatosis type 1 [126] and pancreatic cancer [85,89,127,128] has been documented. In pancreatic cancer, miR-10b is overexpressed in both pancreatic cancer cell lines and tissue samples [127], and is associated with invasive behavior and poor prognosis of pancreatic cancer. MiR-10b decreased the expression of homeobox D10 (HOXD10), a member of the *HOX* genes family, resulting in increased expression of RhoC, a HOXD10 target and a metastasis promoter [36]. Moreover, studies from two different laboratories show an association between the overexpression of miR-10b and urokinase-type plasminogen activator receptor (uPAR), a downstream target of HOXD10 [122,129]. Recently, Korc and coworkers reported that downregulating Tat-interacting protein 30 (TIP30) and upregulating EGFR by miR-10b microRNA caused EGF-mediated invasion in pancreatic cancer [37]. Collectively, miR-10b overexpression could be associated with progression of disease and poor prognosis.

### 3.5. MicroRNA-208 (miR-208)

MiR-208 is located on chromosome 14q11 and is highly expressed in various cancers like esophageal squamous cell carcinoma [39], prostate cancer [130] and hepatocellular carcinoma [131]. Although little information available regarding miR-208 expression and its role as an oncomiR in pancreatic cancer, a recent finding suggests that miR-208 regulates EMT by down-regulating E-cadherin and activating AKT/GSK-3β/snail signaling pathway, thereby promoting tumor cell invasion and metastasis of pancreatic cancer cells [38].

### 3.6. Additional OncomiRs in Pancreatic Cancer

Other oncomiRs contributing to the development and progression of pancreatic cancer include miR-192, which facilitates progression from G0/G1 to S phase by regulating the expression of genes involved in cell cycle control [41,132]. MiR-192 overexpression diminished the expression of p21^Cip1^, p27^Kip1^, p107, p130 and retinoblastoma-1 tumor suppressor protein (Rb1) and increased the expression of cyclin D1, cyclin D2, CDK4, CDC2, and SKP-2 in both pancreatic cancer tissues and cell lines [41,132]. The growth-promoting effect of miR-192 was also documented in colony formation assay and in Panc-1 xenograft tumor model [41]. In addition, miR-192 overexpression attenuated cell apoptosis and stimulated cell proliferation and migration in pancreatic cancer cells [41,132]. In contrast, several studies indicate that miR-192 acts as a tumor suppressor as it inhibits cancer cell proliferation through induction of p53-dependent cell cycle arrest at both the G1 and G2 phases in colon cancer [133] and through targeting Rb1 in lung cancer [40]. OncomiR, miR-424-5p was also found to be overexpressed in pancreatic cancer [42]. This miRNA increased the ability of cells to proliferate, migrate, invade and inhibit cell apoptosis through downregulation of suppressor of cytokine signaling 6 (SOCS6) protein, which leads to elevated ERK1/2 signaling pathway activity [42]. Hao *et al.*, using a miRNA-array differential analysis, reported that miR-483-3p expression was greater in pancreatic cancer tissue compared to adjacent normal tissues [43]. MiR-483-3p overexpression in pancreatic cancer cell lines significantly represses DPC4/Smad4 protein levels and simultaneously promotes cell proliferation and colony formation *in vitro*. In another study, Hao *et al.* found that miR-421 was highly upregulated in specimens of human pancreatic cancer, and it promotes cell proliferation and colony formation by suppressing DPC4/Smad4, a tumor suppressor in pancreatic cancer [44]. Park and colleagues reported that two miRNAs located on chromosome 17p13, miR-132 and miR-212 are highly expressed in PDAC tissues and downregulate the tumor suppressor Rb1, thereby increasing cell proliferation [92]. MiR-191 has been reported to promote pancreatic cancer through targeting UPS10, which suppresses the proliferation and growth of cancer cells by stabilizing the p53 protein [46]. Recently, another miRNA, miR-212, has been established as an oncomiR in PDAC by Ma *et al.* [47]. The researchers reported that this microRNA promotes pancreatic cancer cell proliferation, migration and invasion by targeting the hedgehog signaling pathway receptor patched-1.

## 4. Tumor Suppressor miRNAs (TSmiRs) in Pancreatic Cancer

Unlike oncomiRs, TSmiRs plays a critical role in preventing cancer progression in various organs, including the pancreas, by blocking oncogene function in cancer cells. Several TSmiRs expressions were undetected or minimally detected in pancreatic cancer cells. Enforced expressions of these TSmiRs in cancer cells induce cell growth inhibition and apoptosis. Six of these TsmiRs are described below:

### 4.1. The miRNA-200 Family

**Figure 2:**
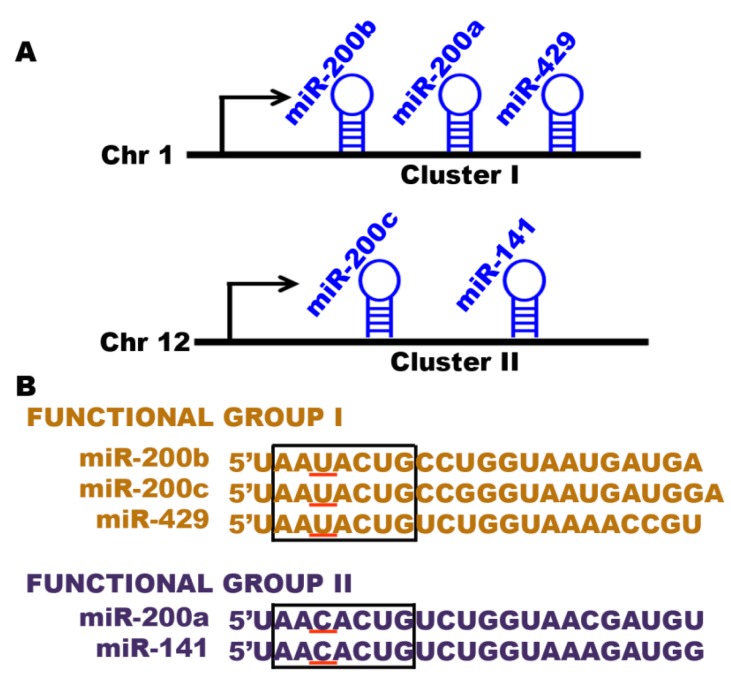
The miR-200 family members. (**A**) The human miR-200 family members are grouped into two clusters: cluster I containing miR-200a, miR-200b, and miR-429 is located on chromosome 1p36, and cluster II contains miR-200c and miR-141, which is located on chromosome 12p13; (**B**) The human miR-200 family members are based on the similarities of their seed sequences (sequence under the box in which one nucleotide is specific for each group (underline)). Members can be divided into two functional groups: functional group I contains miR-200b, -200c, and -429, and functional group II consists of miR-200a and -141, distinguished by a single nucleotide change (U to C).

The miR-200 family consists of five members (miR-200a, miR-200b, miR-200c, miR-141 and miR-429) and can be grouped into two subfamilies based on chromosomal localization and seed sequence [134]. The members of first subfamily are grouped into two clusters: cluster I, containing miR-200a, miR-200b and miR-429, is located on chromosome 1p36, and cluster II contains miR-200c and miR-141, located on chromosome 12p13. The members of second subfamily can be divided into two functional groups: functional group I contains miR-200b, -200c and -429, and functional group II consists of miR-200a and -141. Both share the same seed sequence except one nucleotide (AAUACUG for functional group I and AACACUG for functional group II); thus, they regulate different target genes in cancer (Figure 2) [48,51,52,54]. The tumor suppressive role of the miR-200 family has been reported in several cancers like breast cancer [135,136,137,138,139], renal cancer [140,141,142], colon cancer [143,144], prostate cancer [145,146,147], ovarian cancer [51] and non-small cell lung cancer [148,149]. The aberrant expression of the miR-200 family in pancreatic cancer and its involvement in cancer initiation and progression has been well-demonstrated [25,91,150,151,152,153]. The miR-200 family members have proven to be a key regulator of EMT, a critical step in invasion and metastasis in various cancers [51,134,141,149,151,154]. The transcriptional repressor zinc-finger E-box binding homeobox 1 and 2 (ZEB1 and ZEB2) are crucial inducers of EMT in various cancers and have been shown to promote invasion and metastasis of cancer cells [155,156]. An inverse relation between ZEB1 and the miR-200 family members has been reported to promote EMT and invasion in various cancers, including pancreatic cancer [51,149,150,151,153,157,158,159,160]. The miR-200 family is not only able to inhibit EMT but is also capable of suppressing tumor growth in different xenograft models [147,161,162,163,164,165]. However, a reverse effect of miR-200 family members was reported in colorectal cancer.

Tumor angiogenesis, the formation of new abnormal blood vessels from the existing ones, is a vital, pathological process required for tumor progression and metastasis [166]. Tumor angiogenesis can be blocked by the miR-200 family members via targeting the VEGF signaling pathway components, such as VEGF-A, FLT1/VEGFR1, and KDR/VEGFR2 [49]. Inhibition of VEGFR1 and VEGFR2 by miR-200 family members resulted in the inhibition of tumor growth and angiogenesis in mouse models of PDAC [162]. The overexpression of membrane type-1 matrix metalloproteinase (MT1-MMP) and lesser expression of phosphatase and tensin homolog (PTEN) have been associated with angiogenesis in pancreatic carcinogenesis [167,168]. Interestingly, miR-200c blocks the expression of MT1-MMP with concomitantly increased expression of PTEN in pancreatic cancer cells [152], suggesting that miR200c induced inhibition of tumor angiogenesis could be mediated through the regulation of MT1-MMP and PTEN.

Like other cancers, cancer stem cells play a vital role in tumorigenesis and drug resistance of pancreatic cancer [169]. Several other markers like ABCG2, CD133, ALDH1 and c-Met were identified as CSC/Cancer initiating cell markers [170,171,172]. Interestingly, the miR-200 family members are able to regulate the self-renewal capacity of PC stem cells via regulating multiple gene signatures associated with these events [20,173,174,175].

### 4.2. MicroRNA-34a (miR-34a)

*MiR-34a* gene is located on chromosome 1p36.22 and acts as a potential tumor suppressor in pancreatic cancer [55,56,57] as well as other cancers [176,177,178,179,180]. MiR-34 could be a negative regulator of pancreatic cancer stem cells (PCSCs) growth and survival as CD133+/CD44+ PCSC numbers is decreased by 87% after miR-34 restoration [57]. The activation of mir-34a in pancreatic cancer stem cells by 5-azacytidine (demethylating agent) or SAHA (histone deacetylase inhibitor) resulted inhibition of cell proliferation, cell cycle progression, self-renewal, EMT and invasion with concomitant down-regulation of cyclin D1, CDK4, SIRT1, survivin, Bcl-2, VEGF, and CDK6 and upregulation of p27^Kip1^, P21^Cip1^ and PUMA [59]. Moreover, replacement of miR-34a through systematic delivery greatly suppressed the growth of pancreatic cancer in a xenograft model [181]. Collectively, these studies indicate that the tumor suppressor miR-34a could be a druggable target for pancreatic cancer therapy; however, further studies are warranted.

### 4.3. MicroRNA-146a (miR-146a)

The role of miR-146a can vary in different types of cancers. It can act as an oncomiR in several cancers, including thyroid cancer [182,183], acute promyelocytic leukemia [184] and cervical cancer [185]. Conversely, miR-146a is also a known tumor suppressor in prostate cancer [186], gastric cancer [187,188], breast [189,190], non-small cell lung cancer [191] and pancreatic cancer [192].

### 4.4. MicroRNA-124 (miR-124)

MiR-124 is represented in three genomic loci (miR-124-1 (8p23.1), miR-124-2 (8q12.3) and miR-124-3 (20q13.33)) [67]. MiR-124 has been reported as a tumor suppressor in various cancers including, pancreatic cancer [67,115,193,194,195,196], and interacts with the 3′-UTR of its target, Rac1, to suppress MKK4-JNK-c-Jun-mediated cellular proliferation, invasion and metastasis. Moreover, miR124 known to regulate multiple proliferation-related genes in pancreatic cancer cells such as cyclin-dependent kinase 6 [197,198], Cyclin A2 [199], forkhead box A2 [200] and solute carrier family 16, member 1 (SLC16A1) [197].

Hunt *et al.* showed that miR-124 could also suppress the motility of cancer cells by interacting with 3′-UTR of integrin β1 (ITGB1) [63]. Numerous studies report that miR-124 targets an enhancer of zeste homolog 2 (EZH2), an important transcription factor involved in the proliferation and metastasis of tumor cells [64,66]. MiR-124 is also found to reduce cancer cell aggressiveness by directly targeting rho-kinase2 (ROCK2), an oncogene [64]. Recently, Asuthkar *et al.* examined the inverse relationship between miR-124 and urokinase plasminogen activator (uPA) in pancreatic cancer [201]. They found that uPA is highly expressed while miR-124 is downregulated in pancreatic cancer. Less miR-124 expression results in higher expression of its target LIM homeobox-2 (Lhx2), leading to pancreatic cancer cell stemness.

### 4.5. MicroRNA-203 (miR-203)

MiR-203 is located at chromosome 14q32-33 [68]. MiR-203 was demonstrated to be highly expressed in PDAC samples when compared to normal pancreas tissue and in chronic pancreatitis, and miR-203 overexpression correlated with the worst prognosis in PDAC patients who underwent pancreatectomy [202].

A negative correlation between miR-203 and survivin expression in pancreatic cancer has been recently reported [203]. This study found that miR-203 suppresses the function of survivin by targeting its 3′-UTR and thereby leading to its loss of oncogenic function. Another study also witnesses the tumor suppressive role of miR-203 [69]. Despite discrepancy in the functional role of miR-203, multiple studies suggest that miR-203 prevents PC growth.

### 4.6. Other TSmiRs in Pancreatic Cancer

MiR-615-5p is located within CpG islands of the *HOXC5* gene intron at chromosome 12q13.13 and functions as an outstanding tumor suppressor in hepatocellular carcinoma via inhibition of insulin-like growth factor 2 (IGF-2) [70,71]. Recent studies proved that, compared to adjacent, normal pancreatic tissues, there is significantly lower expression of miR-615-5p in PDAC tissues, as well as an overexpression of miR-615-5p inhibited pancreatic cancer cell proliferation, migration and invasion *in vitro*. MiR-615-5p also inhibited tumor growth and metastasis *in vivo* by targeting 3′-UTR of AKT2, a major downstream effector of phosphatidylinositol3-kinase. Downregulation of miR-615-5p limits its inhibitory effect on IGF-2 and other targets, such as proto-oncogene JUNB, which results in increased tumor growth, invasion and migration capabilities in PDAC cells [73]. miR-206 functions as a tumor suppressor in pancreatic cancer by inhibiting metastatic cell invasion and overall tumor growth and proliferation through the targeting of oncogenic K-Ras, an initiator of PDAC and of ANXA2, a regulator of extra cellular matrix degradation [74]. TSmiR, miR-96 has been reported to inhibit pancreatic cancer cell proliferation, migration and invasion by 3′-UTR of Novel (nua) kinase family 1 (NUAK1), an oncogene in pancreatic cancer [75]. Ectopic expression of miR-96 significantly inhibits malignant behavior of pancreatic cancer cells *in vitro* and *in vivo* by targeting the human ether-a-go-go-related potassium channel (HERG1), whose expression is significantly related to the development of pancreatic cancer [76]. MiR-96 has been shown to inhibit pancreatic cancer cell migration, invasion and growth by targeting both K-Ras and Akt signaling [77]. Recently, Guo *et al.* found that miR-410 acts as a TSmiR which suppresses pancreatic cancer growth, cell invasion, migration and angiogenesis by targeting angiotensin II type 1 receptor (AGTR1) [78]. Tsuda *et al.* demonstrated that miR-3548 inhibits cell division and induce late apoptosis in pancreatic tumor cells by targeting Gli-1 transcription factor, a downstream hedgehog signaling component [79].

## 5. The Therapeutic Potential of MicroRNAs in Pancreatic Cancer

Because of the under- or overexpression of miRNA in cancers, a strong potential for miRNA-based therapeutics exists. Since miRNA exists in all tissues, an aberrant expression of miRNA is not difficult to find. Each miRNA strand has the same effect on the cancer, so using them provides a specific and almost flawless method of combative treatment. In addition, the reintroduction of certain strands does have the ability to increase tumor chemosensitivity [204]. Multiple methods have been experimented on to introduce the miRNA mimic or the anti-miRNA strand into the tumor to restore tumor-suppressor miRNA function or inhibiting the function of oncogenic miRNAs, respectively. Some methods include lipid-based carriers, Cationic polymers, inorganic compounds such as gold and silica-based compounds, and exosomes [204,205,206,207]. Nanovectors can then be created using these carriers which will help transport the miRNA to the right cancer as the nanovector will be coated with specific antibodies [127,208]. Each person afflicted with cancer can treat themselves using customized medicine as a result of the many types of miRNA strands in cancer and the multiple types of delivery systems available [207]. Furthermore, the advancements of nanotechnology could potentially bring new types of delivery systems that both diagnoses and treats a specific types of tumor, and this would greatly increase lifespan and stop the cancer from advancing. Finally, a perfect match is not required for miRNA to bind with mRNA. Only a match between seven consecutive nucleotides is necessary to bind with mRNA, so a safety margin exists, which will help decrease the time needed to create the miRNA mimic or anti-miRNA strand [209].

Recently, therapeutic potential of miRNAs in pancreatic cancer has been demonstrated by a series of *in vitro* and *in vivo* studies. Pramanik *et al.* have synthesized a lipid based nanoparticle for systemic delivery of miR-34a (a tumor suppressor) expression vectors to cancer cells and, by doing so, have revealed the therapeutic value of reestablishing miR-34a expression in orthotopic and subcutaneous pancreatic xenograft models [208]. Furthermore, they also have shown that overexpression of miR-34a may improve the chemotherapeutic effect of traditional chemotherapy agents (e.g., gemcitabine) in pancreatic cancer [208].

MiR-21 overexpression is associated with increased proliferation, invasive properties, gemcitabine chemo-resistance and poor survival in pancreatic cancer patients [85,86,87,88,89,210]. Thus, targeting miR-21 strongly inhibits pancreatic cancer cell proliferation, induces apoptosis and inhibits pancreatic tumor growth in xenograft model [211]. Moreover, targeting miR-21 may stimulate angiogenesis, which may augment tumor drug delivery to enhance the efficacy of gemcitabine. Collectively, it has been shown that inhibition of oncomiRs or re-expression of TS-miRs can sensitize PC cells to the traditional anticancer drugs.

## 6. Pitfalls of miRNA Therapy

Despite having many benefits, pitfalls of miRNA therapy exist. First, miRNA degrades quickly in the blood stream [205]. This occurs as the body believes that the miRNA is an alien and deadly particle, leading to an immune response which kills off the miRNA [205]. Additionally, when the anti-miRNA or miRNA mimic is introduced, the strand may bind to the wrong mRNA which may produce undesired side effects and toxicity [212]. This may occur if the miRNA that was administered into the body creates a better match with another mRNA other than the target mRNA. This comes as a direct drawback from the aforementioned benefit of only requiring a limited amount of nucleotides that needed to be correct. In addition, a certain strand of miRNA may suppress one type of tumor while acting as an oncogene in another tumor. For example, miR-125 acts as a tumor suppressor in breast and bladder cancer, while the same strand of miRNA acts as an oncomiR in pancreatic cancer [207]. This may prove especially difficult when trying to target a specific type of cancer without creating any harsh side effects such as accidently creating another cancer. Finally, despite the advancements in nanotechnology, the creation of nanoparticle delivery systems still remains very costly [205].

## 7. Conclusions

**Figure 3:**
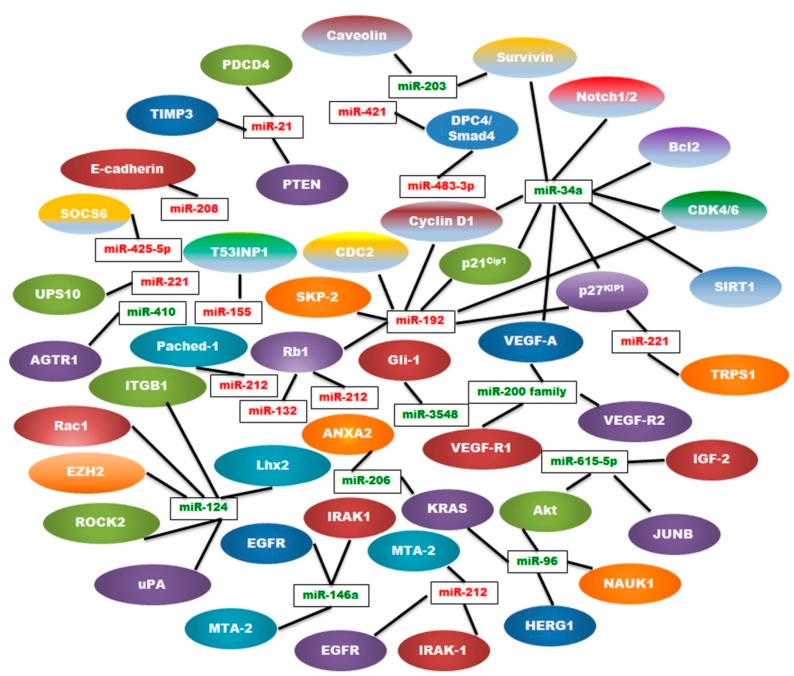
Gene regulatory network of several miRNAs (oncomiRs in red and TSmiRs in green) and their targets in pancreatic cancer.

MicroRNAs represent critical regulators of gene expression and have a wide variety of functions such as tumor cell differentiation, proliferation, cell cycle progression, invasion, and metastasis leading to pancreatic cancer progression. These miRNAs have been described as oncogenes or tumor suppressors (Table 1 and Table 2, Figure 3) and many of them are used for diagnosis and as prognostic or predictive tools. Using this data, miRNAs can be used as therapeutic agents to combat pancreatic cancer. However, transportation methods for the miRNAs may induce toxicity, so more research is necessary to understand the effects of introducing a carrier into the body. Further work to identify downstream targets of these miRNAs will bring greater comprehension regarding their mode of action and shine a fresh light on using miRNAs clinically as biomarkers or therapeutic targets to combat pancreatic cancer.

## Figures and Tables

**Table 1:** Different oncogenic miRNAs in pancreatic cancer.

MiRNA	Type	Regulation	Location	Targets	References
*miR-21*	Oncogenic	Up	17q23.2	PTEN, PDCD4, TIMP3	[9,25,26,27]
*miR-221*	Oncogenic	Up	Xp11.3	TRPS1, p27^kip1^	[28,29,30,31]
*miR-155*	Oncogenic	Up	21q21	TP53INP1, SOCS1	[24,32,33,34]
*miR-10b*	Oncogenic	Up	2q31.1	TIP30	[35,36,37]
*miR-208*	Oncogenic	Up	14q11	E-cadherin	[38,39]
*miR-192*	Oncogenic	Up	11q13.1	Rb1, p27^Kip1^, p21^Cip1^, CyclinD1/2, SKP-2,CDK4, CDC2	[40,41]
*miR-425-5p*	Oncogenic	Up	3p21.31	SOCS6	[42]
*miR-483-3p*	Oncogenic	Up	11p15.5	DPC4/Smad4	[43]
*miR-421*	Oncogenic	Up	Xq13.2	DPC4/Smad4	[44]
*miR-132*	Oncogenic	Up	17p13	Rb1	[45]
*miR-212*	Oncogenic	Up	17p13	Rb1	[45]
*miR-191*	Oncogenic	Up	3p21.31	UPS10	[46]
*miR-212*	Oncogenic	Up	17p13.3	Patched-1	[47]

**Table 2:** Different Tumor suppressor miRNAs in pancreatic cancer.

MiRNA	Type	Regulation	Location	Targets	References
*miR-200 Family*	Tumor Suppressor	Down	1p36, 12p12	VEGF-A, FLT1/VEGFR1 KDR/VEGFR2	[48,49,50,51,52,53,54]
*miR-34a*	Tumor Suppressor	Down	1p36.22	Notch1/2, Bcl-2, Cyclin D1, Survivin, SIRT1,VEGF, CDK4/6, p27^KIP1^	[55,56,57,58,59]
*miR-146a*	Tumor Suppressor	Down	5q33.3	EGFR, IRAK-1, MTA-2	[60,61,62]
*miR-124*	Tumor Suppressor	Down	8p23.1, 8q12.3, 20q13.33	Rac1, ITGB1, EZH2, ROCK2, uPA, Lhx2	[63,64,65,66,67]
*miR-203*	Tumor Suppressor	Down	14q32-33	Survivin, Caveolin‑1	[68,69]
*miR-615-5p*	Tumor Suppressor	Down	12q13.13	Akt2, IGF2, JUNB	[70,71,72,73]
*miR-206*	Tumor suppressor	Down	6p12.2	K-Ras, ANXA2	[74]
*miR-96*	Tumor Suppressor	Down	7q32.2	NUAK1, Akt, HERG1, K-Ras	[75,76,77]
*miR-410*	Tumor Suppressor	Down	14q32.31	AGTR1	[78]
*miR-3548*	Tumor Suppressor	Down	-	Gli-1	[79]
